# Plasma Testosterone and Androstenedione Levels Follow the Same Sex-Specific Patterns in the Two *Pan* Species

**DOI:** 10.3390/biology11091275

**Published:** 2022-08-27

**Authors:** Ruth Sonnweber, Jeroen M. G. Stevens, Gottfried Hohmann, Tobias Deschner, Verena Behringer

**Affiliations:** 1 Department of Behavioral and Cognitive Biology, University of Vienna, Djerassiplatz 1, 1030 Vienna, Austria; 2 Behavioral Ecology and Ecophysiology, Department of Biology, University of Antwerp, Campus Drie Eiken, Building D, D1.21, Universiteitsplein 1, 2610 Antwerp, Belgium; 3 Max Planck Institute for Evolutionary Anthropology, Deutscher Platz 6, 04103 Leipzig, Germany; 4 Max Planck Institute of Animal Behavior, Am Obstberg 1, 78315 Radolfzell/Konstanz, Germany; 5 Comparative BioCognition, Institute of Cognitive Science, University of Osnabrück, Artilleriestrasse 34, 49090 Osnabrück, Germany; 6 Endocrinology Laboratory, German Primate Center, Leibniz Institute for Primate Research, Kellnerweg 4, 37077 Göttingen, Germany

**Keywords:** androgens, female aggression, dominance, chimpanzee, *Pan troglodytes*, bonobo, *Pan paniscus*

## Abstract

**Simple Summary:**

Generally male mammals are more aggressive than their female peers. In these males, aggressive behavior is linked to levels of androgens; higher levels of testosterone are predictive of higher aggression rates or more severe aggression. There are some species where the pattern of sex-specific aggression is reversed, and it was hypothesized that high levels of androgens may be responsible for social dominance and aggressiveness in these females. Studies so far found that females of species with sex-reversed aggression patterns (e.g., spotted hyenas and ring-tailed lemurs) had lower plasma testosterone levels than their male peers, but a precursor of testosterone, androstenedione, was comparable or even higher in females than in males. This supported the idea that selection for female aggressiveness may be facilitated through augmented androgen secretion. Here we show that in two sister species, bonobos and chimpanzees, that differ in terms of sex-specific aggression patterns, females have lower plasma testosterone levels and higher plasma androstenedione levels than their male peers. Thus, our data do not support a theory of a role of female androgen levels on the expression of sex-specific patterns of aggression.

**Abstract:**

In most animals, males are considered more aggressive, in terms of frequency and intensity of aggressive behaviors, than their female peers. However, in several species this widespread male-biased aggression pattern is either extenuated, absent, or even sex-reversed. Studies investigating potential neuro-physiological mechanisms driving the selection for female aggression in these species have revealed an important, but not exclusive role of androgens in the expression of the observed sex-specific behavioral patterns. Two very closely related mammalian species that markedly differ in the expression and degree of sex-specific aggression are the two *Pan* species, where the chimpanzee societies are male-dominated while in bonobos sex-biased aggression patterns are alleviated. Using liquid chromatography–mass spectrometry (LC-MS) methods, we measured levels of plasma testosterone and androstenedione levels in male and female zoo-housed bonobos (N = 21; 12 females, 9 males) and chimpanzees (N = 41; 27 females, 14 males). Our results show comparable absolute and relative intersexual patterns of blood androgen levels in both species of *Pan*. Plasma testosterone levels were higher in males (bonobos: females: average 0.53 ± 0.30 ng/mL; males 6.70 ± 2.93 ng/mL; chimpanzees: females: average 0.40 ± 0.23 ng/mL; males 5.84 ± 3.63 ng/mL) and plasma androstenedione levels were higher in females of either species (bonobos: females: average 1.83 ± 0.87 ng/mL; males 1.13 ± 0.44 ng/mL; chimpanzees: females: average 1.84 ± 0.92 ng/mL; males 1.22 ± 0.55 ng/mL). The latter result speaks against a role of androstenedione in the mediation of heightened female aggression, as had been suggested based on studies in other mammal species where females are dominant and show high levels of female aggressiveness.

## 1. Introduction

Darwin [1], was the first to attribute male dominance over females to male intrasexual selection. Males have higher potential reproductive output than females and consequently competition for agonistic power is more intense among males than among females [2]. This favors the selection of morphological, physiological, and behavioral traits that increase agonistic dominance in males, particularly. On the behavioral level, most males show higher rates of aggressive behaviors and allocate more energy and time to aggressive interactions than females (e.g., [3]). Thus, there is a widespread male-biased sexual dimorphism in the number of aggressive behaviors shown and, associated with this, in the evolution of morphological traits aiding in aggressive encounters (e.g., large body size, large canines, or weaponry, [1]). However, there are mammalian species where females are more, or at least as aggressive as their male peers [4], where the typical sexual dimorphism in body size is not expressed (first described in [5]), and/or where male dominance over females is absent. To better understand the selection for aggression in females of these species, or the counter-selection of aggression in males, the proximate physiological mechanisms driving female aggression can be investigated. A good starting point for this is to investigate the physiological differences between males and females in species with enhanced male aggression. One set of the physiological markers that has received a lot of attention in this respect are androgen levels, including testosterone and one of its precursors, androstenedione. These steroid hormones play an important part in the ontogeny of male behavior, including aggression [6], and in the organization, activation, and expression of aggressive behavior in adult males [7,8].

There are several female-dominated mammalian species, where all females dominate all males and in which females aggress males as much, or even more, than males aggress females. The potential physiological underpinnings of this phenomenon are well studied in a number of these species (e.g., spotted hyenas, several species of lemurs, marmosets and tamarins, a variety of rodent species such as the naked mole rats or Syrian hamsters, and rock hyraxes; for a review see [3]). For instance, in spotted hyenas (*Crocuta crocuta*) females show higher aggression rates than their male peers and blood testosterone levels are higher in males than in females [9,10,11], but androstenedione levels are comparable between the sexes [9,10]. In ring-tailed lemurs (*Lemur catta*), androstenedione to testosterone ratios are higher in females than in males [12], which suggests that in this primate species androstenedione may also constitute a crucial precursor hormone for androgenic effects on female aggression [3]. Overall, studies on various mammalian species where male aggression rates are equal or lower compared to female aggression rates indicate that androgens play an important, although not exclusive part in the observed dimorphism of these behavioral patterns (reviewed in [3]). Our understanding of the association between female aggression and the activating (and organizing) role of androgenic steroids is far from complete and is limited to species where the sexual dimorphism in aggression rates is pronounced [3]. 

One species that departs from the mammalian norm regarding sex-specific patterns in aggressive behavior and sex-biased dominance relations, although less pronounced than the afore mentioned species, is the bonobo (*Pan paniscus*). While bonobos are often seen as non-aggressive, aggressive behavior does occur both in the wild and in captivity. Female bonobos aggress males (but less so than for instance spotted hyenas), in particular in coalition with other females, and are considered to be co-dominant with males [13,14], which, by definition, requires that not all individuals of one sex completely dominate all individuals of the other sex [15]. A study on personality in 44 zoo-housed bonobos found no sex differences in amount of aggression given, but found that males received more aggression, compared to females [16]. This co-dominance of the sexes, and the absence of male-biased aggression is one of the most pronounced contrasts of bonobos to their congeners the chimpanzees (*Pan troglodytes*), where generally all adult males dominate all adult females [17]. In captive chimpanzee groups, aggression is far more frequent between males than females [18]. Moreover, aggression in chimpanzees is considered to be more severe than in bonobos [19,20]. While in the wild, the overall frequency of aggression is about twice as high in male chimpanzees as compared to male bonobos [21], female bonobo aggression rates are higher or comparable to rates observed in female chimpanzees [13]. These differences are also reflected in lower sexual dimorphism in bonobos. While typically male bonobos are heavier and have larger canines than females, differences in body size and canine size are small compared to chimpanzees [22,23,24]. While comparative data on interspecific differences in rates of aggression in chimpanzees and bonobos remain scarce, the available data so far show that the two species differ in sex-specific distribution of aggression, which leads to the question; how these reported differences in aggression translate into species-specific sex-differences in androgen levels? 

So far, measurements of urinary and salivary testosterone levels in samples from zoo-housed bonobos and chimpanzees have revealed lower testosterone (metabolites) levels in male bonobos compared to male chimpanzees, overlapping levels in male and female bonobos, and a pronounced sex dimorphism in chimpanzees’ testosterone (metabolites) levels [25,26]. Male to female ratios in urinary testosterone metabolites are highly skewed towards males in chimpanzees, but relatively balanced in bonobos [25]. This finding seems to be mainly driven by high urinary testosterone levels in male chimpanzees, rather than elevated urinary testosterone levels in female bonobos. These physiological patterns appear biologically meaningful given that it has been suggested that testosterone modulates aggression [27,28], and seems to fit with data on sex-specific aggression rates and aggression severity in the two species [19,20,29,30,31]. However, the results are rather puzzling for four reasons: First, no relationship between testosterone levels and female aggressiveness has been found for species where the sex-specific patterns in aggression rates are strongly biased towards females [3,10,12,32]. Rather androstenedione levels were higher or at least comparable between the sexes in these species. Secondly, male bonobos have large testes [33], and steep male hierarchies, which seem to predict reproductive success [34,35], just as it is the case in chimpanzees [36]. As these traits are largely testosterone-driven, it can be predicted that testosterone levels should be considerably higher in male than in female bonobos, similar to what is found in chimpanzees. Third, ontogenetic evaluation suggests no functional differences in urinary testosterone between the two *Pan* species ([37], but see [26]). Finally, methodological concerns limit the strength of the two studies delivering results on species-specific patterns in androgen levels in the two sexes: (i) The study by Sannen and colleagues [25], is based on urine samples analyzed using an enzyme-immuno-assay. In urine, testosterone is highly metabolized, and therefore, measurements of such samples refer to metabolites instead of concentrations of the native hormone. While those metabolite measurements correlate well with blood testosterone levels, they are not as specific as measurements of the native hormone as can be obtained from plasma as well as saliva samples [38,39]. (ii) Wobber and colleagues [26], collected saliva with cotton swabs and used enzyme immune assays to measure hormone levels from these samples. Cotton contains plant substances that are able to bind on the antibody of an enzyme immune assay, thereby artificially increasing testosterone measurements [40,41,42]. This effect can be avoided by using the technique of liquid chromatography-mass spectrometry, which separates compounds included in a sample by polarity and differences in mass/charge ratios [43], and is therefore able to differentiate between testosterone and the molecularly similar plant substances. 

These four considerations warrant further examination of sex-specific patterns in testosterone and androstenedione levels in the two *Pan* species (for an overview of potential scenarios see Table 1). We measured plasma testosterone and androstenedione levels of female and male zoo-housed bonobos and chimpanzees. If co-dominance of the sexes in bonobos is mediated by female aggressiveness via testosterone or androstenedione levels (hypothesis 1, Table 1) we would expect the following pattern of species-specific plasma testosterone and androstenedione levels in the sexes: (i) In female bonobos, levels of both hormones should be closer to levels of males than is the case in chimpanzees. Female bonobo levels could be lower, not significantly different, or even higher than male levels. (ii) In chimpanzees, females should show considerably lower levels than males. If this is the case and if male levels are comparable in the two *Pan* species, then (iii) female bonobos might have higher levels than female chimpanzees. The second scenario would be that male bonobos show particularly low levels of testosterone and/or androstenedione compared to chimpanzee males as female alliance formation prevents them to monopolize mating partners through aggression (hypothesis 2, Table 1). In this case we would (i) expect a smaller female to male plasma testosterone and/or androstenedione ratio in bonobos than in chimpanzees, (ii) distinctively lower levels of female plasma testosterone and/or androstenedione than in male levels in chimpanzees, but (iii) lower plasma testosterone and/or androstenedione levels in male bonobo than in male chimpanzees, while females are expected to have comparable plasma testosterone and/or androstenedione levels across the two species. Alternatively, males of either species may express higher testosterone and/or androstenedione levels than their respective female peers (hypothesis 3, Table 1), as would be suggested by large testes in males of both species, steep male rank hierarchies, and a functionally corresponding pubertal development of male bonobos and chimpanzees.

## 2. Materials and Methods

### 2.1. Ethics

Blood sample collection and associated procedures were undertaken by zoo veterinarians and ethically reviewed and approved by the respective zoo authorities. Apes were never anaesthetized for the purpose of this study. All blood samples used in this study were obtained for management reasons (e.g., health checks, implantation of contraception, and transfer to other facilities). We adhered to the ASP Principles for Ethical Treatment of Non-Human Primates. All procedures were in accordance with relevant national guidelines for the care and use of laboratory animals. We conformed to the Directive 2010/63/EU and complied with the ARRIVE guidelines. The Animal Ethics and Experimentation Board of the Faculty of Life Sciences of the University of Vienna reviewed and approved the methods and procedures used in this study (ethics approval number 2022-009).

### 2.2. Animals and Sample Collection

We analyzed plasma testosterone and androstenedione levels in 62 blood samples of 21 bonobos (12 females, 9 males) and 41 chimpanzees (27 females, 14 males) housed in 13 different zoos. Management of the populations was comparable across zoos. As testosterone levels in both *Pan* species increase with puberty [in bonobos see [37], in chimpanzees (see [44]), we included only samples from individuals older than ten years of age in our sample. The bonobos in our sample were between 11 and 39 years of age (median age: females 21 years, males 18 years; life expectancy in bonobos is about 52 years in captivity; data on wild populations are very scarce), chimpanzees were between 10 and 49 years old (median age: females 19 years, males 29 years; life expectancy in chimpanzees is about 45 in the wild and 57 years in captivity, although reports on individuals older than 60 years of age in captivity exist). Ten female chimpanzees (out of 27) but no female bonobos (out of 12) were on contraceptives. All apes lived in social groups and were fed with fruits and vegetables several times daily. Fresh water was available *ad libitum* at all zoos. 

Samples were collected, between 1996 and 2016, by veterinarians of the respective zoos during routine husbandry practices (either health checks or transfers between zoos). Apes were sedated when samples were taken. Blood samples were centrifuged, and the supernatant was transferred to a plastic vial. Samples were labelled with the date of sample collection and the individual’s name and stored at minus 20 °C at the zoo until transported frozen to the Max Planck Institute for Evolutionary Anthropology (MPI-EVA). Alternatively, samples were shipped to the MPI-EVA immediately after collection and centrifuged there. All samples remained frozen until they were thawed for hormone measurement (no thawing-freeze cycles in between). At the MPI-EVA, all further procedures and analyses were conducted between 2016 and 2019. For thawing, the samples were taken out of the minus 20 °C walk-in freezer and brought to the extraction laboratory. The laboratory was kept at a constant room temperature of 21 °C. As soon as the samples were thawed, the extraction procedure commenced.

### 2.3. Sample Extraction and Analytical Methods

Testosterone and androstenedione were extracted from plasma samples following [39,45], with minor modifications as described in [46]. Each plasma sample was mixed with 400 µL acetonitrile and 5 µL internal standard (a mixture described in [47]). Extracts were transferred into high-performance liquid chromatography (HPLC) vial inserts and stored at minus 20 °C until measurement. Plasma androgen levels were measured using liquid chromatography tandem mass spectrometry with a Waters Acquity UPLC separation module equipped with a binary solvent manager, sample manager, and a column oven (Waters, Milford, MA, USA). Separation was performed on a reverse phase C-18 column (Acquity UPLC BEH C18 1.7 µm, 2.1 × 100 mm) protected by an in-line filter unit. The composition of eluent A and B was water containing 0.1% formic acid, and acetonitrile, respectively. The gradient was 25% B (0–1.5 min), linear increase to 65% B (1.5–8.5 min), 95% B (8.5–10 min), and 25% B (10–12 min). Flow rate was 0.35 mL/minute. 10 µL of the extract were injected. Mass spectrometric analyses were carried out on a Xevo TQ-S tandem quadrupole mass spectrometer (Waters, Milford, MA, USA) with electrospray ionization (ESI) in positive mode. We excluded three samples that had a deviation of internal standard recovery of more than ± 50% from the expected values from our analysis and not enough volume to repeat the measurement. We quantified data with MassLynx (Version 4.1; TargetLynx-Software, Waters Corporation, Milford, MA, USA). The limit of quantification for testosterone and androstenedione was 0.02 ng/mL. Plasma androgen levels are expressed in ng/mL.

### 2.4. Statistical Analyses

To assess sex- and species-specific patterns of plasma testosterone and androstenedione levels in the two *Pan* species, we fitted a Linear Mixed Model with a Gaussian error structure [48,49]. To reduce the skewness in the data and achieve a more normal distribution, we log-transformed testosterone and androstenedione levels, which were entered as response variables into two separate models. As we expected that sex differences in these androgen levels might differ between bonobos and chimpanzees (see Table 1), we included the two-way interaction term of species and sex as a predictor in the model. To account for age-related variation in androgen levels (e.g., [50,51]), we added individual age as a control variable (a table showing the number of individuals by sex and zoo and age is attached in the Supplementary Material S1). As some females in our dataset were on contraceptives and contraceptives have been shown to affect plasma androgen levels (e.g., [52,53,54]), we added contraception as a control variable. Predictor and control variables were z-transformed (centered to a mean of zero with a standard deviation of one) to obtain comparable estimates [55]. As samples of individuals came from different zoos and site-specific factors might have effects on androgen levels, we included a random intercept for the zoo an individual was housed at.

To check for potential issues with multicollinearity between individual predictors, we tested the variance inflation factor (VIFs) of our standard linear model (function “vif” of the R package “car”, [56], but found no indication for collinearity (maximum VIF = 1.283 in the testosterone model; maximum VIF = 1.283 in the androstenedione model). Likelihood ratio tests were used to compare competing models with each other (full model vs null model, and reduced models) [57]. Quantile–quantile plots and distribution of residuals plotted against fitted values were inspected to check model assumptions. Models were fitted in RStudio, Version 4.0.2 (RStudio Team (2020). RStudio: Integrated Development for R. RStudio, PBC, Boston, MA, USA, URL http://www.rstudio.com/ (accessed on 26 July 2022)), using the function “lmer” (R package “lme4”, [58]). 

## 3. Results

In Table 2, an overview of the basic statistical descriptive values (mean ± standard deviation, median, and ranges) for plasma testosterone and plasma androstenedione in male and female bonobos and chimpanzees are given.

### 3.1. Plasma Testosterone

**Figure 1:**
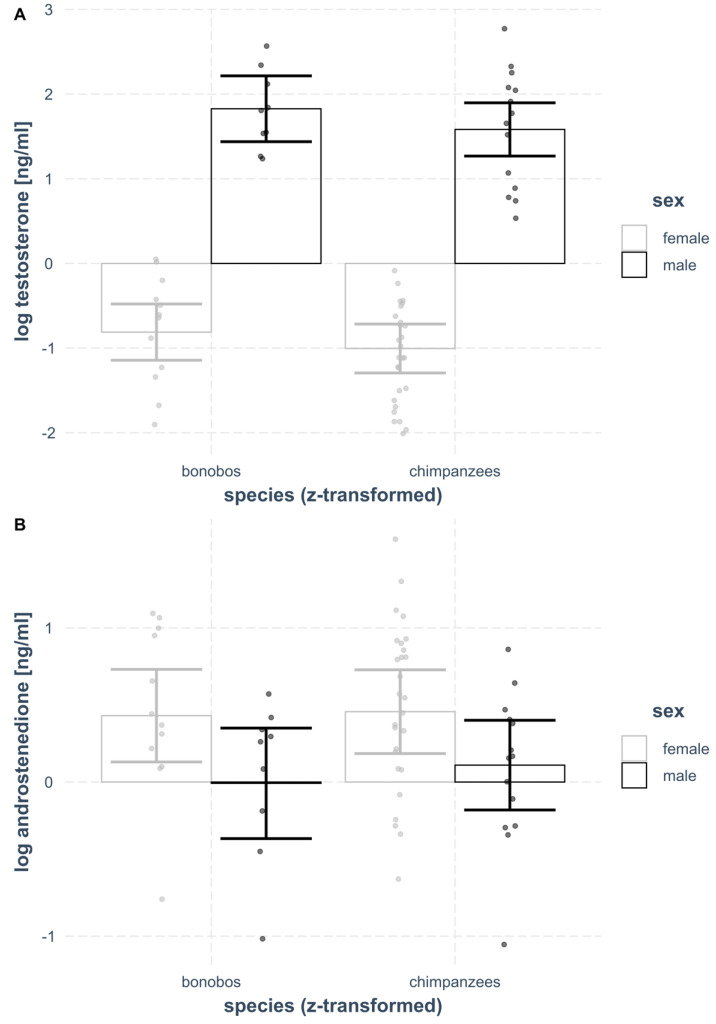
Bonobo and chimpanzee (**A**) plasma testosterone and (**B**) plasma androstenedione levels by species and sex. (**A**) The upper plot shows that females (light grey bars) of either species (bonobos on the left, chimpanzees on the right of the plot) had significantly lower plasma testosterone levels (log-transformed values on the y-axis) than males (black bars) of the respective species. (**B**) Female plasma androstenedione levels were higher than male levels in both Pan species, as depicted in the lower plot.

Comparing the full model (including the two-way interaction between species and sex) and the null model (including solely the control variable of individual age and the random intercept for zoo), revealed that they were significantly different from each other (λ^2^ = 102.51, df = 3, *p* < 0.001). Therefore, we proceeded reducing model complexity by eliminating the two-way interaction and only keeping main effects of variables in the model. This showed that the full (containing the interaction term) and the reduced model (containing only main effects) were not significantly different from each other (λ^2^ = 0.024, df = 1, *p* = 0.878). The simpler model (reduced model) was the final model (see Table 3), revealing that sex (estimate ± SE = 2.610 ± 0.161, *p* < 0.001, see Figure 1A), but not species significantly predicted plasma testosterone levels. Male plasma testosterone levels were higher than female plasma testosterone levels in both *Pan* species.

### 3.2. Plasma Androstenedione

The full model (including the two-way interaction between species and sex) and the null model (including solely the control variable of individual age and the random intercept for zoo) differed from each other with a *p*-value close to significance (λ^2^ = 7.13, df = 3, *p* = 0.068). Reducing model complexity by eliminating the two-way interaction, keeping main effects of variables in the model revealed that the full (containing the interaction term) and the reduced model (containing only main effects) were not significantly different from each other (λ^2^ = 0.11, df = 1, *p* = 0.741). Thus, the simpler model (reduced model) was the final model (see Table 4), revealing that sex (estimate ± SE = −0.39 ± 0.14, *p* = 0.008, see Figure 1B), but not species significantly predicted plasma androstenedione levels; females of both species had higher plasma androstenedione levels than males.

## 4. Discussion

In this study, we investigated the plasma levels of two androgens, testosterone and androstenedione, in zoo-housed bonobos and chimpanzees. We found that both androgens followed the same sex-specific patterns in both species. Male bonobos and chimpanzees had higher plasma testosterone levels than their female peers, with similar sex-specific levels of plasma testosterone across the two species. This suggests that the higher levels of female aggressiveness in bonobos compared to chimpanzees [13], in particular in relation to the level of aggressiveness in the respective males of the two species [30,59], are not associated with sex-specific levels in plasma testosterone or androstenedione. 

Our results thus contradict previous findings based on urinary and salivary measures of testosterone that showed a sexual dimorphism in testosterone levels in chimpanzees, but not in bonobos [25,26]. These contrasting results are likely caused by methodological issues, such as differences in laboratory methods and extraction procedures, and/or differences in the matrices collected for hormone measurements (as discussed in the introduction). Similar issues have also been reported and discussed by Goymann et al. [10] who compared results generated by different studies in spotted hyenas.

Androgen levels represented in our dataset were collected from zoo-housed apes. It can be argued that these levels are not representative for the species in the wild, and it can be questioned if they are physiologically meaningful. In zoo populations, male–male competition is certainly reduced when compared to wild populations, in particular in the case of chimpanzees, for instance, because only few males are housed together in one group. Furthermore, zoo-housed apes often show less locomotion compared to wild conspecifics [60], which will likely result in a smaller muscle mass as compared to free living individuals. Since both high levels of competition and increased muscle mass are associated with higher testosterone levels, at least in males [61], data collected on wild populations of the two *Pan* species might have revealed different results to our study on zoo-housed individuals. Of course, it is hardly possible to collect blood samples from healthy wild living apes that would then allow to draw conclusions about the generality of our results. However, we can compare our data with plasma androgen measurements from chimpanzees living at Ngamba Island, a chimpanzee sanctuary in Uganda where individuals live in larger groups and larger enclosures than the zoo-housed chimpanzees in our study. Plasma samples from individuals of this population were analyzed using LC-MS [39,45], which allows us to compare hormone levels between this and our study. Plasma testosterone levels of those sanctuary chimpanzees fall in the lower range of our measurements. This slightly lower plasma testosterone level in the sanctuary population might reflect a trade-off between competitive strength through testosterone and its energetic costs [61]. Plasma androstenedione levels in the zoo-housed chimpanzees of our study are three times higher than those measured in the sanctuary chimpanzees. Another indication that our results are physiologically meaningful comes from comparisons of androgen sex ratios measured in our study and data derived from humans. Our testosterone and androstenedione levels and ratios are comparable to levels measured in women and men (e.g., [62]). In human blood, testosterone levels measured with LC-MS/MS, are 14 to 18 times higher in men than in women [62,63], similar to our results, where male chimpanzees had 15 times higher plasma testosterone levels than females, and bonobo males had 13 times higher plasma testosterone levels than their female peers. Androstenedione levels measured with LC-MS are 61% higher in women than in men [62], but see [64] for nearly equal androstenedione levels in the two sexes in humans]. This is comparable to our results that show 60% higher plasma androstenedione levels in male bonobos and 70% higher plasma androstenedione levels in male chimpanzees than in their female peers. While we still cannot rule out that plasma androgen levels from wild populations of great apes may still differ dramatically from those derived from zoo-housed populations, the above-mentioned comparisons to sanctuary-housed apes and humans give confidence in the general validity of our findings.

Another aspect that may have affected testosterone levels in our study subjects is the fact that samples were drawn while individuals were sedated; in some species some types of sedation and anesthesia were associated with lower (e.g., [65]) or increased (e.g., [66]) testosterone level measurements during and after sedation. In other studies, no relation between testosterone levels and anesthesia/sedation drugs (ketamine/xylazine) were found (e.g., [67,68]). To our knowledge, the effects of ketamine/xylazine, which are commonly used by zoo veterinarians for great apes, or any other anesthesia drug (such as barbiturates) have not yet been investigated in bonobos or chimpanzees. Therefore, it is difficult to assess the potential influences of sedation/anesthesia on our blood testosterone measurements. However, since all individuals represented in our study, independent of species, were anaesthetized when blood samples were drawn, the anesthesia effect should be constant across all samples. 

Our results are unexpected when considering that bonobos have a less pronounced physical sexual dimorphism than chimpanzees [22,23,24], and males of the two *Pan* species differ in terms of the intensity of mate competition and the benefits derived from such interactions [31,69], which led to speculation that adult male chimpanzees generally would have higher testosterone levels than male bonobos; a pattern that was confirmed in the earlier testosterone measurements from urine and saliva samples [25,26,70] (potential problems with these measurements are discussed above). Our blood testosterone measurements did not confirm this prediction and blood testosterone levels did not differ between males of the two species. As already suggested by the lack of a correlation between urinary androgens and aggressive behaviors in a small group of zoo-housed bonobos [71], the connection between aggressive behavior and androgens may not lie in circulating testosterone levels, but in the potential species differences in testosterone binding affinity to testosterone receptors, as well as the density of testosterone receptors in specific brain areas, such as hypothalamic neurons or the amygdala (e.g., [72,73,74,75]). If male bonobos were found to have more testosterone receptors in the relevant brain areas than male chimpanzees, we could argue that more testosterone molecules would need to bind to receptors in bonobos to elicit the same behavior as in chimpanzees. Another venue for this kind of research is to further look into androgen receptor polymorphisms, where differences between the two *Pan* species have already been revealed [76,77]. 

On the other hand, our results are in line with studies on other mammalian species where female testosterone levels were not higher compared to males, despite a high degree of female aggressiveness and female dominance [3,10,12,32]. Likewise, in polyandrous bird species, where females have a higher mating competition than males, no sex-reversal in testosterone levels was found [78,79,80,81]. One interpretation of our findings is that sex-differences in plasma testosterone levels reflect the strength of intrasexual competition among males, but do not relate to rates or intensity of female–female or female–male aggression. Thus, together, these studies indicate that testosterone levels are not crucial in mediating the frequency and/or intensity of aggression in females. Again, as suggested above for interspecies differences, differences between the sexes in androgen receptor density and distribution could mediate differences in behavior, as suggested for instance in a study on a bird species with reversed sex roles (*Centropus grillii*). In this species, females express a higher density of mRNA for androgen receptors in the brain areas controlling social behaviors, as compared to their male conspecifics, suggesting that in this case, sex differences in behavior may be mediated by sex-specific neuroendocrine cascades, rather than hormone levels [82]. For great apes such data still need to be presented.

The second candidate androgen that was considered to play a role in female aggressiveness and dominance is androstenedione. Like in spotted hyenas [10], and ring-tailed lemurs (e.g., [32]), female bonobos in our study had higher plasma androstenedione levels than male bonobos. This fits with the idea that androstenedione is related to female aggression as female bonobos aggress males and other females [13], while males do not show high levels of aggression towards females as they do not seem to gain from intersexual aggression [83]. However, we also found significantly higher plasma androstenedione levels in female chimpanzees compared to males, despite a clear male-biased pattern in aggression rates and intensity [19,20,29,31,83]. Female chimpanzees do not aggress males [84], and while they were reported to show severe aggression towards other females [85], generally aggression rates among females are rather low [86]. Furthermore, female chimpanzees can form alliances against male aggression [87]. Thus, high levels of androstenedione in female chimpanzees are not likely to be related to inter- or intra-sexual aggression. Therefore, at least in the two *Pan* species, androstenedione does not seem to explain sex-specific expressions in aggressive or dominance relations. Interestingly, in women, also no relationship between androstenedione and physical aggression was found, but women with higher androstenedione levels were more likely to express competitive feelings verbally [88]. 

There are various reports about intraspecific aggression in both *Pan* species (e.g., [16,18,21,83]) and the findings of these studies have been incorporated into evolutionary models of human aggression (e.g., [29,59,89]). However, comparative data on the rate and nature of aggression remain scarce, rendering the information status of this topic preliminary. Therefore, we encourage future studies to take a comparative approach to compare aggression rates and the potential mechanistic role of androgens in male and female bonobos and chimpanzees. 

Besides the two androgens considered here, other hormones, such as dehydroepiandrosterone (DHEA) [8,90,91], progesterone [92], and vasopressin [93,94,95], could play a role in modulating female aggression. In particular, DHEA is suggested to be important for the expression of aggressive behavior, especially when testosterone levels are low [8], as is the case in the females in our study. Alternatively, co-dominance in bonobos may not be achieved by female aggression against males, but rather by using leverage through female social bonds [83,96]. Here, the circulating levels and/or receptor genes and distribution of vasopressin or oxytocin could be interesting makers to examine. These hormones have been associated with social bonding and have been found to have different receptor distribution in the brains of bonobos and chimpanzees, which suggests functional differences of these hormones in the two *Pan* species [97]. For example, oxytocin receptors are found in reward regions of the human brain, but not in chimpanzees [98], and there are differences in allelic variants of oxytocin receptor genes between the two *Pan* species [99]. Vasopressin receptor gene 1a varies between bonobos and chimpanzees, which is potentially linked to interspecies behavioral differences [100]. Therefore, tackling these hormonal markers in the context of dominance relations between the sexes may be an exciting avenue for future research.

Despite the many species differences that we observe in bonobos and chimpanzees and the resulting implications for interpretations of human evolution [29,101], the two species may not be as drastically different in every aspect as often portrayed [102]. Previously we reported that the two sister species show no ontogenetic differences in urinary testosterone excretion patterns [37]. Our present results reveal that also among adult individuals the absolute and relative intersexual patterns of blood androgen levels are similar in the female–male co-dominated societies of bonobos and in the male-dominated structures of chimpanzees. Moreover, these patterns found in the two *Pan* species are similar to patterns found in humans, suggesting that they may have deep evolutionary roots, independent of the role of aggression in mediating social dominance relationships within each of the three species.

## 5. Conclusions

Our results, and the conclusions we can draw from them, highlight the importance of comparative work. By comparing blood androgen levels of individuals of both sexes of two closely related species that differ markedly in the trait of sex-specific aggressiveness, we can understand if these hormones may have a role in mediating the difference in these behaviors. Our results do not indicate a role of androstenedione or testosterone in the mediation of female aggression in the two *Pan* species. In both species we found similar patterns of intersexual blood androgen levels, both in absolute and relative terms. While plasma testosterone levels were higher in males in bonobos and chimpanzees, plasma androstenedione levels were higher in females of either species. This speaks against suppositions that resulted from earlier studies on mammalian species where females are described as more aggressive than their male peers. 

## Figures and Tables

**Table 1:** Predictions for female plasma androgen (testosterone androstenedione) levels in relation to male plasma androgen levels in bonobos and chimpanzees.

Predictions of Sex Differences in Plasma Androgen (A) Levels in the Two *Pan* Species	Within Species Bonobos	Within Species Chimpanzees	Species Comparison (Bonobo, b vs. Chimpanzee, c)
Sex-specific aggression patterns are mediated by female androgen levels (Hypothesis 1)	♀ < or = or > ♂, because ♀ A are high	♀ < ♂	b♂ = c♂ b♀ > c♀
Sex-specific aggression patterns are mediated by male androgen levels (Hypothesis 2)	♀ = or > ♂, because ♂ A is low	♀ < ♂	b♂ < c♂ b♀ = c♀
Males need high androgen levels for agonistic power and to ensure reproductive success, while androgens play a lesser role in females (Hypothesis 3)	♀ < ♂	♀ < ♂	b♂ = c♂ b♀ = c♀

♀ = female, ♂ = male, b = bonobo, c = chimpanzee.

**Table 2:** Mean, median, and ranges of female and male bonobo and chimpanzee androgens. Sex ratios of mean testosterone and androstenedione in bonobos and chimpanzees are calculated.

Androgen		Bonobo		Chimpanzee	
Testosterone (ng/mL)		Female	Male	Female	Male
	Mean ± SD	0.5 ±0.3	6.7 ± 2.9	0.4 ± 0.2	5.8 ± 3.6
	Median	0.5	5.9	0.3	5.7
	Range	0.2–1.1	3.7–12.1	0.1–1.0	1.57–14.50
	Sex-ratio	12.6		14.6	
**Androstenedione (ng/mL)**					
	Mean ± SD	1.8 ± 0.9	1.1 ± 0.4	1.8 ± 0.9	1.2 ± 0.6
	Median	1.6	1.2	1.7	1.2
	Range	0.5–3.2	0.4–1.7	0.6–4.5	0.4–2.4
	Sex-ratio	0.6		0.7	

**Table 3:** Results of the final testosterone model. The model contained the main effects of species (bonobos vs chimpanzees), sex (female vs male), the age of the subject at the time of sample collection, whether an individual was on contraceptives, and a random intercept for zoo. Estimates, standard errors, and *p*-values for individual variables are shown. Reference categories are indicated for categorical variables.

Results of the Final Testosterone Model	Estimate	Standard Error	*p*-Value
Intercept	−0.807	0.145	
Species (z-transformed): bonobo	−0.215	0.165	0.205
Sex (z-transformed): female	2.610	0.161	<0.001
Individual age (z-transformed)	0.074	0.175	
Contraception (z-transformed): no contraception	−0.195	0.226	
	Variance	Standard deviation	
Intercept zoo	0.009	0.095	
Residual variance	0.314	0.561	

**Table 4:** Results of the final androstenedione model. The model contained the main effects of species (bonobos vs chimpanzees), sex (female vs male), the age of the subject at the time of sample collection, whether an individual was on contraceptives, and a random intercept for zoo. Estimates, standard errors, and *p*-values for individual variables are shown. Reference categories are indicated for categorical variables.

Results of the Final Androstenedione Model	Estimate	Standard Error	*p*-Value
Intercept	0.414	0.134	
Species (z-transformed): bonobo	0.063	0.152	0.692
Sex (z-transformed): female	−0.386	0.142	0.008
Individual age (z-transformed)	−0.033	0.153	
Contraception (z-transformed): no contraception	0.020	0.201	
	**Variance**	**Standard deviation**	
Intercept zoo	0.026	0.161	
Residual variance	0.227	0.476	

## Data Availability

The datasets generated during and/or analyzed during the current study are available from the corresponding author on reasonable request.

## Supplementary Materials

The following supporting information can be downloaded at: https://www.mdpi.com/article/10.3390/biology11091275/s1, Table S1: Overview of number of males/females per age and zoo.
