# Supplementary material for: Plasma Testosterone and Androstenedione Levels Follow the Same Sex-Specific Patterns in the Two Pan Species
Source: Biology (Basel). 2022 Aug 27;11(9):1275. doi: 10.3390/biology11091275 (PMC9495489; doi:10.3390/biology11091275)
Supplement: Supplementary file 1 [file biology-11-01275-s001.zip › biology-1826441-supplementary.pdf]

Table S1: Number of individuals by age and zoo. The upper table shows the number of females per age and zoo. The lower table shows the number of males per age and zoo.

FEMALES	BONOBOS								1 Total	CHIMPANZEES								2 Total	Grand Total
**age**	**Berlin**	**Cologne**	**Frankfurt**	**Leipzig**	**Planckendae**	**Stuttgart**	**Twycross**	**Wuppertal**		**Bremerhave**	**Gossau**	**Leipzig**	**Lisboa**	**Magdeburg**	**Munich**	**Stuttgart**	**Twycross**		
11							1		1	1						1		2	3
12						1			1							1		1	2
13												1						1	1
14												2	1		1	1	1	6	6
15											1					1		2	2
16												1						1	1
18				1					1										1
19			1		1				2			1						1	3
20		1							1										1
22					1				1										1
23																	1	1	1
24														1				1	1
25								1	1							1		1	2
26												1						1	1
27																	1	1	1
29																	1	1	1
30					1				1			1						1	2
34																1		1	1
35										1								1	1
36	1								1								1	1	2
37						1			1							1		1	2
39		1							1								1	1	2
49																	1	1	1
**Grand Total**	**1**	**2**	**1**	**1**	**3**	**2**	**1**	**1**	**12**	**2**	**1**	**7**	**1**	**1**	**1**	**7**	**7**	**27**	**39**

MALES	BONOBOS				1 Total	CHIMPANZEES							2 Total	Grand Total
**age**	**Berlin**	**Cologne**	**Planckendae**	**Twycross**		**Bremerhave**	**Gossau**	**Leipzig**	**Lisboa**	**Munich**	**Stuttgart**	**Twycross**		
10								1					1	1
11			2		2									2
12						1				1			2	2
15	1		1		2									2
18				1	1									1
19										1		1	2	2
22			1		1									1
28									1				1	1
29											1	1	2	2
30												2	2	2
31		1			1									1
33												1	1	1
34				1	1									1
35											1		1	1
36												1	1	1
37		1			1									1
38							1						1	1
**Grand Total**	**1**	**2**	**4**	**2**	**9**	**1**	**1**	**1**	**1**	**2**	**2**	**6**	**14**	**23**
